# Assessment of the trophic state of the Soła River dam cascade, Polish Carpathians: a comparison of the methodology

**DOI:** 10.1038/s41598-023-33040-2

**Published:** 2023-04-11

**Authors:** Ewa Jachniak, Andrzej Jaguś

**Affiliations:** Department of Environmental Protection and Engineering, University of Bielsko-Biala, Willowa 2, 43-300 Bielsko-Biała, Poland

**Keywords:** Environmental sciences, Limnology

## Abstract

The aim of this research was to determine the trophic state of mountain dam reservoirs, which are characterized by greater hydrological and ecological dynamics than lowland reservoirs. The trophic state of three dam reservoirs forming a cascade system was investigated. Trophic evaluation was carried out based on multiple criteria, i.e., (1) the content of chlorophyll *a* in the water, (2) planktonic algal biomass, (3) groups and species of algae, (4) the total phosphorus concentration in the water, and (5) the Integral Trophic State index (ITS). The analyzed parameters were characterized by high variability during the study period, which to a large extent may have resulted from the mountain environmental conditions. The greatest dynamics concerned parameters related to phytoplankton development. Unequivocal determinations of the trophic states of the reservoirs were difficult; however, it was found that in successive reservoirs of the cascade (from the highest to the lowest), a reduction in water fertility occurred.

## Introduction

Dam water reservoirs fulfill various functions within water-economic systems, e.g., as reservoirs for human water supply, for agricultural irrigation, for recreational and leisure purposes, and to supply industries, and they often have the potential to be multifunctional^1^. In most cases, for a reservoir to fulfill its functions properly, it must contain high-quality water. It is therefore necessary to monitor the parameters of the reservoir water^2,3^. Identifying the trophic state of a reservoir is a very practical measure that is used for the comprehensive assessment of water quality in a reservoir^4,5^. Trophy reflects the abilities of the entire geosystem of the reservoir for biological production^6^, which includes food abundance, often called fertility. There are four main trophic states: oligotrophic (lowest fertility), mesotrophic, eutrophic and hypertrophic (highest fertility). A trophic state usually shows the long-term trends in the quality of a reservoir, so it can be the basis for the formulation of protective and recultivation programs.

The trophy of inland waters, including dam reservoirs, can be determined by various methods. The methods usually consist of the analysis of the physical and chemical parameters of water or of the biological parameters of living organisms^7–10^. For water, the following parameters are most often used: concentration of total phosphorus and phosphates, concentration of total nitrogen and nitrates, concentration of dissolved oxygen and chlorophyll *a*, as well as the pH, conductivity and transparency of water (Secchi disk visibility). The features of the various organisms also indicate the trophic state—macroinvertebrates, ichthyofauna, macrophytes, and phytoplankton (planktonic algae). A worldwide method for trophy classification has been proposed for example by the Organization for Economic Co-operation and Development—OECD^11^ and by the European Environment Agency—EEA^12^.

This study focused in large part on parameters related to phytoplankton development^13–21^, although in mountain reservoirs, this development may be disturbed by various environmental factors, especially temperature drops and frequent flood waves^22–25^. As Wilk-Woźniak^20^ writes, a rapid restoration of phytoplankton communities follows floods (within a period of approximately a week), with the dominant forms being those that prefer high turbulence and low water transparency.

This study investigates the identification of the trophy of dam reservoirs in the Polish Carpathians. The dam reservoirs in this region are a very valuable element of the environment due to the lack of natural lakes that are comprehensively used. The reservoir water is usually intended for supplying the human population, as well as some industrial sectors. The reservoirs are therefore of strategic importance and should be intimately monitored and protected^26^. The state organizations in Poland that monitor the quality of surface water do not determine the trophic state of the reservoirs; therefore, scientific studies are needed. The mountain reservoirs are characterized by lower hydrological and ecological stability than lowland reservoirs; therefore, trophic evaluation should be carried out in a multidirectional manner.

**Figure 1:**
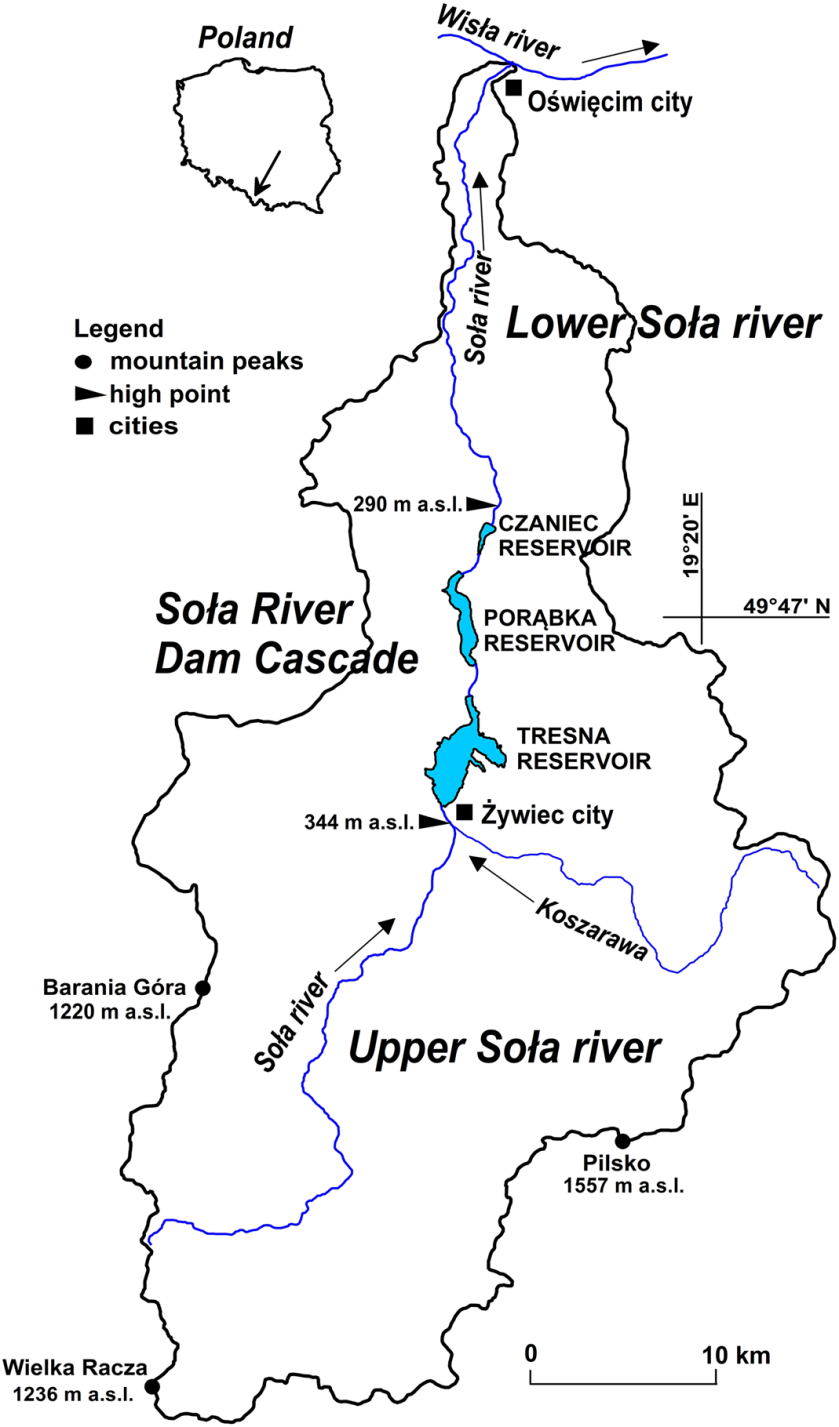
Soła River drainage basin with the researched reservoirs.

Three dam reservoirs were selected for this study—Tresna (49° 43′ N, 19° 12′ E), Porąbka (49° 47′ N, 19° 12′ E) and Czaniec (49° 50′ N, 19° 13′ E), which form the so-called cascade of the Soła River^27^. They were built in the Polish Carpathians in the middle course of the Soła River (Fig. 1) during a period from the 1930s to the 1960s.

The aim of this study to attempt to define the trophic state of Soła cascade dam reservoirs on the basis of the features of phytoplankton that live in the water, as well as selected chemical parameters of the water. The following assessment criteria were applied: the content of chlorophyll *a* in the water, planktonic algal biomass, groups and species of algae, the total phosphorus concentration in the water, and the Integral Trophic State index, which reflects the relationship between the pH of water and the oxygen saturation of water. It was hypothetically assumed that the trophic state of the reservoirs is variable due to their location in the mountain catchment area. The researchers’ intention was also recognition whether the trophy analysis carried out with different methods (criteria) will show the same results or different results. In this article the results of trophy assessment according to the applied criteria were compared. The practical aspect of the research relied on identify of water quality parameters, whose values reach the level of eutrophy, to indicate actions was possible, which reduce the influx of pollutants from specific sources. It is particularly important due to the water supply purpose of the Czaniec Reservoir and the recreational use of Tresna and Porąbka Reservoirs.

The research was conducted in 2019 year. Previous publications about water quality in the Soła cascade show the research results of a selective (fragmentary) character. Planktonic algae research in all reservoirs was conducted in the 1960s and 1970s by Bombówna and Bucka^28^, Kyselowa and Krzeczkowska-Wołoszyn^29^ and Paluch et al.^30^. These studies were continued in the 1990s by Stachowicz and Czernoch^31^. Research of water chemistry of the Tresna and Porąbka Reservoirs was also conducted, especially by the State Environmental Protection Service, but also scientists [e.g.,^30,32^]. Chemical composition of Czaniec Reservoir water was examined mainly by the owner of the water intakes, due to water supply function of this reservoir. Stachowicz and Czernoch^31^ 30 years ago made an attempt to evaluate the trophy of the Tresna and Porąbka Reservoirs, but without taking into account any trophicity scale (any limit values of the parameters). In the twenty-first century the trophy of reservoirs was evaluated more precisely, but still selectively^33–35^. The research described in this article is the first comprehensive research on the trophy of the Soła cascade, which includes all reservoirs at the same time and which uses several criteria of the trophic evaluation.

## Cascade of the Soła River characteristics

The Soła River is one of the main rivers in the Polish Carpathians. The average decline in the river from its source to the Tresna Reservoir is 0.98%. The catchment area to the dam of the Czaniec Reservoir has a mountainous character. Its area is 1119.16 km^2^. According to our cartographic analyses, the average elevation of this area is approximately 650 m a.s.l., while the average decline is approximately 23%. Over 70% of the catchment area declines by more than 20%, and in approximately 20% of the catchment area, the land declines by more than 30%. Due to these conditions, the Soła River is characterized by very high variability of flows and violent flood phenomena. As Jaguś^36^ calculated for the area of the inflow to the Tresna Reservoir, the annual flows of the Soła (in the multiyear period 1956–2015) were as follows: minimum from 0.59 to 3.04 m^3^∙s^–1^, average from 9.08 to 27.87 m^3^ s^–1^ and maximum from 92.6 to 1250 m^3^ s^–1^.

**Figure 2:**
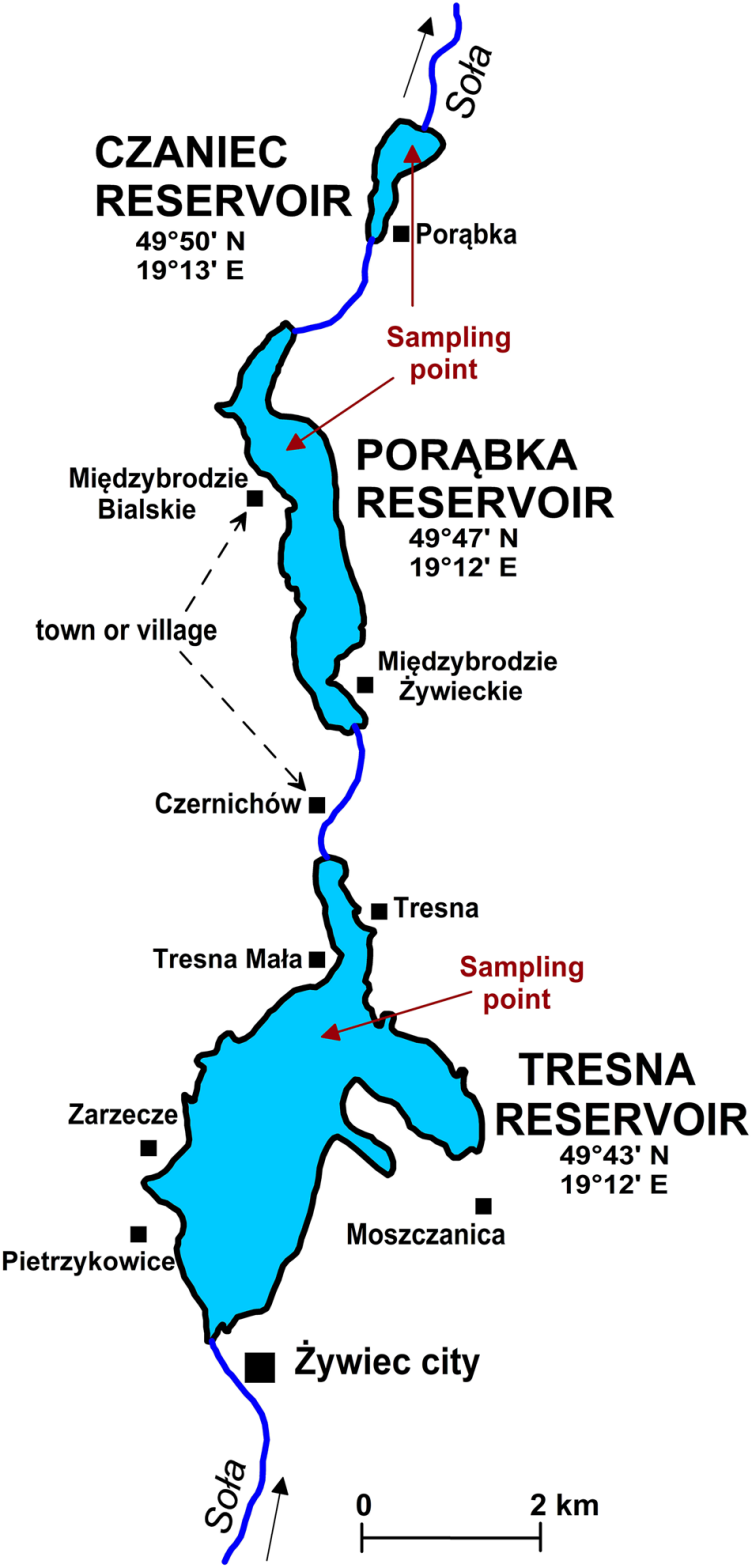
Soła River dam cascade—situational sketch with sampling points.

The cascade is a very interesting research object because the individual reservoirs differ from each other in terms of morphometric (Table 1, Fig. 2), hydrological and hydrobiological parameters.

**Table 1:** Characteristics of reservoirs based on data in the monograph of the Soła River dam cascade^27^.

The feature of the reservoir	Tresna Reservoir	Porąbka Reservoir	Czaniec Reservoir
Year of handover to exploitation	1967	1938	1967
Minimum water table level [m a.s.l.]	328.36	311.09	295.36
Maximum water table level [m a.s.l.]	344.86	321.49	298.06
Total capacity [mln m^3^]	98.11	27.19	1.32
Maximum reservoir area [ha]	967	333	54
Maximum depth [m]	28.0	19.0	5.5
Average depth [m]	9.9	8.2	2.4
The lenght of reservoir [km]	6.25	6.50	1.70
The length of the coastline [km]	33.7	15.2	4.9

The first reservoir of the cascade—Tresna—is situated in a natural mountain cirque. It has a retention function, i.e., it reduces the risk of flooding. It is also used recreationally, although it is considered the most polluted reservoir of the cascade^34^. The reservoir is powered by the water of the Soła River and by the water of the smaller rivers and streams. The catchment area of the reservoir is 1036.91 km^2^. The water in the reservoir is changed on average 5.88 times during the year, that is, the water change time on average is 62 days^31^.

The Porąbka Reservoir is located in the gorge section of the Soła River by a mountain range; therefore, it has the form of an elongated gutter. It has its own catchment area with a surface of 55.01 km^2^; in addition, it receives the water from the Soła River that flows out of the Tresna Reservoir. The Porąbka Reservoir has two main functions: first, it secures water resources for a hydroelectric power station, and second, it is a recreational area. The water in the reservoir is changed on average 20 times per year, that is, every 18 days^31^.

The Czaniec Reservoir is located in the area where the Soła River flows out of the mountain gorge; therefore, it is shallow and embanked. This reservoir has its own catchment area with a surface area of 27.24 km^2^, from which mountain streams with high erosion potential flow. However, the debris brought into the reservoir does not remain in the reservoir due to the very rapid rate of water change. The water is changed on average every 0.73 days, which is 500 times per year^31^. The Czaniec Reservoir is inaccessible to the public because it serves as a drinking water supply. There are water intakes within it.

These brief characteristics of the reservoirs indicate that each of them can be treated as a separate geosystem with different conditions for the development of phytoplankton. The reservoirs are connected by one water retention management system, but the only system connecting these reservoirs in terms of nature is the Soła River. A section of the river’s free flow between the Tresna and Porąbka Reservoirs has a length of approx. 1.5 km, while that between the Porąbka and Czaniec Reservoirs, it is approximately 1.2 km.

## Research protocols and methods for determining trophic state

### Water sampling and scope of analysis

The water from the reservoirs was analyzed during a period from May to October 2019. This period corresponds to the production cycle of phytoplankton. The six-monthly measurement series were realized. A total of 36 water samples were collected from the reservoirs (6 months × 3 reservoirs × 2 nearby positions). The dates of water sampling from the reservoirs depended on the hydrological conditions. Samples were collected during the rainless periods, when the flows had stabilized for at least a week and the water did not contain visible mineral suspensions. This allowed for the omission of short-term extreme conditions related to flood situations. The points of water collection were selected such that they were not subject to the influence of flowing rivers and streams and shore zones (Fig. 2). These places were located at the possible high depths and in calm waters. The water was collected by a boat using a bathometer with a capacity of 5 dm^3^; the samples were collected from a depth of 1 m.

After sample collection, the water pH and oxygen saturation of the water were immediately determined. Then, the water samples were transported under refrigerated conditions to the water laboratory at the University of Bielsko-Biala. The concentration of total phosphorus, the content of chlorophyll *a* and features of phytoplankton were analyzed in the laboratory.

### Chlorophyll a

To determining chlorophyll *a,* which is water insoluble, the water (one dm^3^) was filtered and the seston with phytoplankton was obtained^37^. Whatman glass fiber filters (type GF/A, diameter 1.6 μm) were used for this purpose. From the filtered and ground material, chlorophyll was extracted by using a solution of acetone and distilled water (9 ml acetone and 1 ml distilled water). The extraction time was 10–15 min during grinding and 24 h during storage in a refrigerator. The chlorophyll extract was analyzed spectrophotometrically. The wavelengths 663 nm and 750 nm and 665 nm and 750 nm were used to measure the absorbance of the obtained extracts. The concentration of chlorophyll *a* was calculated in accordance with the Polish standard PN-86/C-5560.02 (formula). The final results were given in µg∙dm^–3^, with an accuracy of 0.01.

1 $$X = \frac{{26,73 \cdot \left[ {\left( {A_{663b} - A_{750b} } \right) - \left( {A_{665a} - A_{750a} } \right)} \right] \cdot V_{E} }}{{V_{w} \cdot l}}$$

*X*—concentration of the chlorophyll *a* [µg∙dm^–3^], *A*_*663b*_, *A*_*750b*_—the absorbances of the extract, which were determined before adding of hydrochloric acid [0, 12 mol∙dm^–3^], *A*_*665a*_, *A*_*750a*_—the absorbances of the extract, which were determined after adding of hydrochloric acid, *V*_*E*_—volume of the prepared extract [dm^3^], *V*_*W*_—volume of the filtered water [dm^3^], *l*—thickness of the cuvette absorbing layer [cm].

Trophic state was determined based on the concentration of chlorophyll *a* in the water. According to OECD classification^11^, the limit values of the concentrations are as follows:

- below 2.5 µg∙dm^–3^ – oligotrophy;
- from 2.5 to 8 µg∙dm^–3^—mesotrophy;
- from 8 to 25 µg∙dm^–3^—eutrophy;
- above 25 µg∙dm^–3^—hypertrophy.

### Quantitative and qualitative features of phytoplankton

The analyses of the phytoplankton biomass required sample preparation. The phytoplankton samples were concentrated by decanting and sedimentation (over 48 h) to 1 dm^3^ and then up to 100 cm^3^. If the sludge size of the 100 cm^3^ compacted sample was greater than 0.2 cm^3^, the sample was then concentrated to 10 cm^3^. Conversely, if the sludge size was less than 0.2 cm^3^, the sample was compacted to 5 cm^3^. During compaction (in dark glass bottles), the samples were preserved with Lugol liquid at 30 drops per dm^3^^38^.

The amount of biomass and the species composition of the phytoplankton were determined based on microscopic observations^39^. A Nikon Eclipse 200 light microscope was used. The single cells, cenobium or colonies were accepted as a unit (specimen) of the phytoplankton, while in filamentous algae, the length of the filament of 100 μm was treated as a unit (specimen) of the phytoplankton. A chamber (type of Kolkwitz) with a height of 0.4 mm and a diameter of 20 mm was used for the analysis. The algae were calculated in 15–27 view fields in three repetitions.

The phytoplankton biomass (given in wet mass) was calculated by comparing the phytoplankton organisms to geometric figures^40^. The trophic state was determined based on the phytoplankton biomass according to the classification, which was elaborated by Heinonen^41^:

- from 0.14 to 0.68 mg∙dm^–3^—oligotrophy;
- from 0.69 to 1.20 mg∙dm^–3^—transitional state (oligo/mesotrophy);
- from 1.21 to 1.98 mg∙dm^–3^—mesotrophy;
- from 1.99 to 3.44 mg∙dm^–3^—transitional state (meso/eutrophy);
- from 3.45 to 6.93 mg∙dm^–3^—eutrophy;
- from 6.94 to 17.49 mg∙dm^–3^—transitional state (eu/hypertrophy);
- 17,50 and more mg∙dm^–3^—hypertrophy.

Determination of species composition was based on the appropriate keys^42–45^. Eight groups of phytoplankton organisms were distinguished during analyses, and their share in each water sample was calculated. They were the following groups: *Bacillariophyceae* (Engl. Diatoms), *Chlorophyceae* (Engl. Green Algae), *Cryptophyceae* (Engl. Cryptophytes), *Chrysophyceae* (Engl. Chrysophytes), *Euglenophyceae* (Engl. Euglenophytes), *Dinophyceae* (Engl. Dinoflagellates), *Conjugatophyceae* (Engl. Desmids), *Cyanobacteria* (Engl. Blue‒Green Algae). The occurrence of certain groups, especially algal species, can serve as an indicator of trophic state. This is detailed in a following portion of this study.

### Total phosphorus

The total phosphorus (P) in the water samples was determined according to the Polish Standards PN-EN 1189:2000. This method was based on the mineralization of the sample with sulfuric and nitric acids and then a color reaction with ascorbic and molybdic acids. The final stage involved spectrophotometric measurement at a wavelength of 880 nm, with an accuracy of 0.001 mg∙dm^–3^.

The concentration of total phosphorus in the water indicates a specified trophic state. According to the OECD classification^11^, the limit values of the total phosphorus concentration for individual trophic states are as follows:

- below 0.01 mg∙dm^–3^—oligotrophy;
- from 0.01 to 0.035 mg∙dm^–3^—mesotrophy;
- from 0.035 to 0.1 mg∙dm^–3^—eutrophy;
- above 0.1 mg∙dm^–3^—hypertrophy.

### pH and oxygen saturation: Integral Trophic State index (ITS)

The water pH (potentiometric method) and oxygen saturation (measurement with a microcomputer oxygen meter, %O_2_) were measured immediately after collection of the water samples. Both parameters were based on an integral criterion of trophic state developed by Neverowa-Dziopak^4,46^ in the form of the Integral Trophic State index (ITS). ITS reflects the production and decomposition balance of organic substances, which reveals itself in the gas economy of the water. ITS can be used when there is a linear relationship between pH values and the oxygen saturation of water. After performing a correlation analysis between the pH and oxygen saturation values, ITS was calculated according to the formula:

2 $$ITS = \frac{{\Sigma \left( {pH} \right)}}{n} + a\left[ {100 - \frac{{\Sigma \left( {\% O_{2} } \right)}}{n}} \right]$$

*n*—number of measurements, *a*—empiric coefficient (linear regression coefficient).

The trophic state was determined based on calculated ITS values, according to the following scale^4,46^:

- ITS below 6.0—dystrophy,
- ITS from 6.0 to 6.6—ultraoligotrophy,
- ITS from 6.7 to 7.3—oligotrophy,
- ITS from 7.4 to 8.0—mesotrophy,
- ITS above 8.0—eutrophy.

## Results and discussion

During this study, it was hypothesized that the eutrophication process (the increase in fertility) of the Soła cascade would be most visible in the Tresna Reservoir; conversely, it was predicted that the Czaniec Reservoir would be characterized by the lowest fertility. Pollutants and debris material flow into the Tresna Reservoir from a catchment area of 1036.91 km^2^. This catchment has a small retention capacity, which results largely from improper land development by the human population^36,47^. A major problem is the significant siltation of the bowl of this reservoir, especially in the inflow area of the Soła River^48^. According to Łajczak^49^, the Tresna Reservoir retains 91% of the debris that flows into this reservoir. It is exposed to the mixing of water and bottom sediments during floods, which causes high turbidity in the water under such situations. On the other hand, the Tresna Reservoir has a purifying effect on the water of the Soła River—the purifying effect of dam reservoirs, especially cascade systems, has been described in publications from various countries [e.g.,^23,50^]. Stachowicz and Czernoch^31^ found that the waters of the Porąbka and Czaniec reservoirs have more favorable physical and chemical parameters than the water of the Tresna Reservoir.

The reservoirs of the Soła cascade, especially Tresna and Porąbka receive all flood waves. In addition, the reservoirs function in the mountain area with dynamic meteorological conditions. In the Tresna and Porąbka reservoirs there is stratification in summer and natural mixing of waters in spring and autumn. It all affects the accumulation of nutrients in bottom sediments and recirculation to water^51,52^. Therefore, it was expected that the trophic state of the reservoirs won’t be constant during the research period. This supposition was confirmed by the results of the research, which are described below.

Water samples for research were collected during the stable hydrometeorological conditions, when there were no factors disturbing the development of phytoplankton. However, it is worth noting that not all months fulfilled the criterion of the normal period in terms of the precipitation amount. The values of the Standardized Precipitation Index (SPI) prove it, which were calculated on the basis of the available multi-year precipitation data (2008–2022) for the nearby Radziechowy Meteorological Station (data of the Polish Meteorological Service—IMGW). The classification of individual months according to SPI during the research is as follows: May—wet (0.95), June—extremely dry (− 2.25), July—dry (− 0.93), August—normal (0.46), September—normal (− 0.22), October—normal (0.30).

### Chlorophyll a

**Figure 3:**
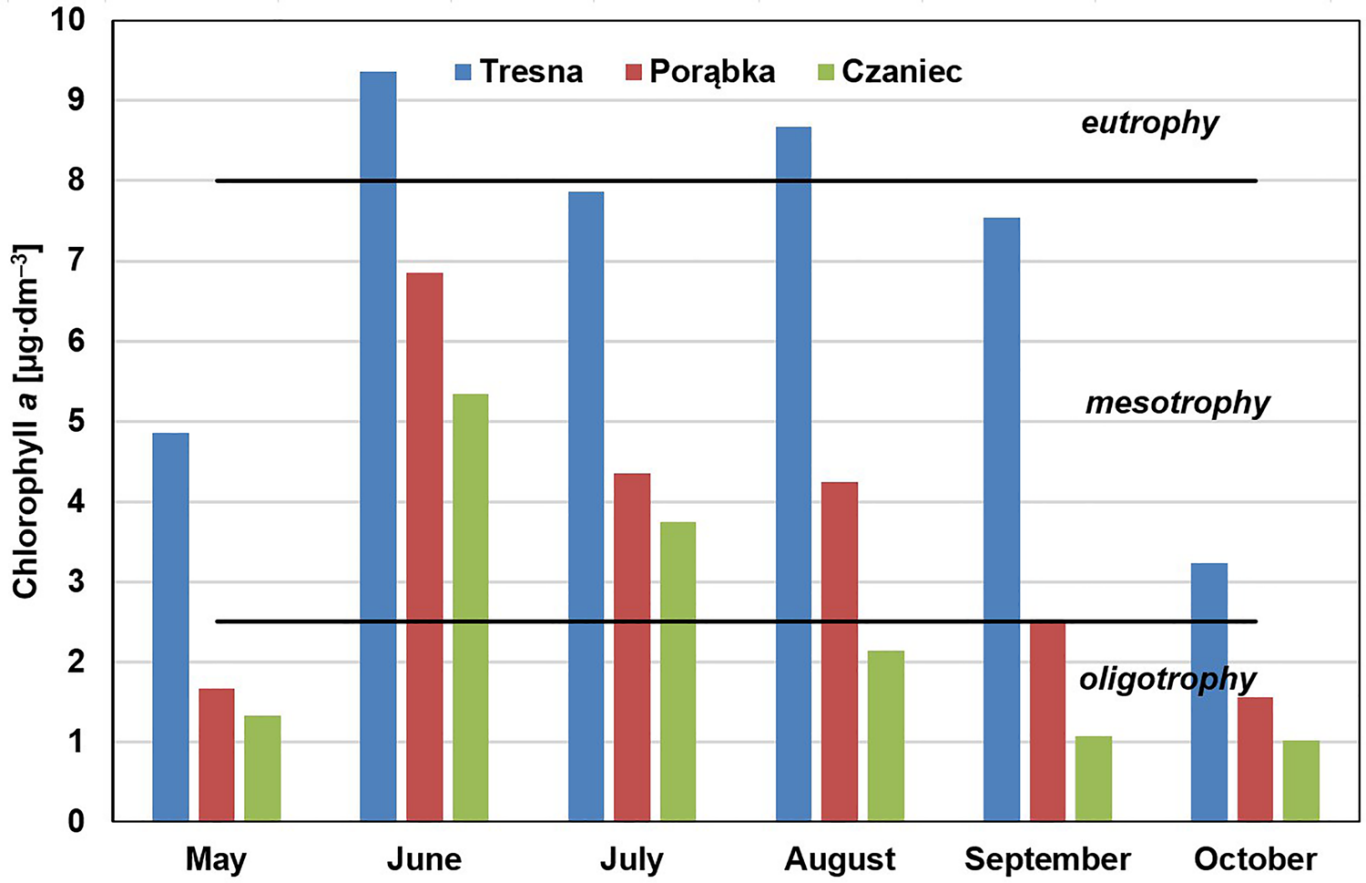
Concentrations of chlorophyll *a* in water of the Soła cascade reservoirs in 2019 year and the OECD trophic classification^11^.

Chlorophyll *a* is a basic color components of plants and algae and is practically insoluble in water. Its content in water is an indicator of phytoplankton content and of the intensity of the photosynthesis process^53,54^. The content of chlorophyll *a* in the water of the Soła cascade reservoirs was very diverse. The results of the research are shown in Fig. 3. The highest concentrations of chlorophyll *a* in each series of measurements were recorded in the waters of the Tresna Reservoir (maximum 9.36 µg∙dm^–3^), followed by the waters of the Porąbka Reservoir (maximum 6.85 µg∙dm^–3^), and then the waters of the Czaniec Reservoir (maximum 5.34 µg∙dm^–3^). Chlorophyll *a* concentrations also varied during the study period (Tresna: SD = 2.17; Porąbka: SD = 1.85; Czaniec: SD = 1.60); the greatest concentration occurred in June, and the lowest occurred in October. High concentrations of chlorophyll *a* were maintained from June to September in the Tresna Reservoir, which indicates intensive biological production during the entire summer period.

The average concentration of chlorophyll *a* in the Tresna Reservoir was equal to 6.92 µg∙dm^–3^, so in the range of mesotrophy, but in two series of measurements, the concentration of chlorophyll *a* was in the eutrophic range. The Porąbka Reservoir in the four measurement series was classified at the mesotrophy level, while in the two-series, it was classified at the oligotrophy level. According to the average value (3.53 µg∙dm^–3^), it could be defined as mesotrophic. In the case of the Czaniec Reservoir, the average concentration of chlorophyll *a* (2.44 µg∙dm^–3^) indicated oligotrophy, but in June and July, mesotrophic levels were found.

### Planktonic algae biomass

**Figure 4:**
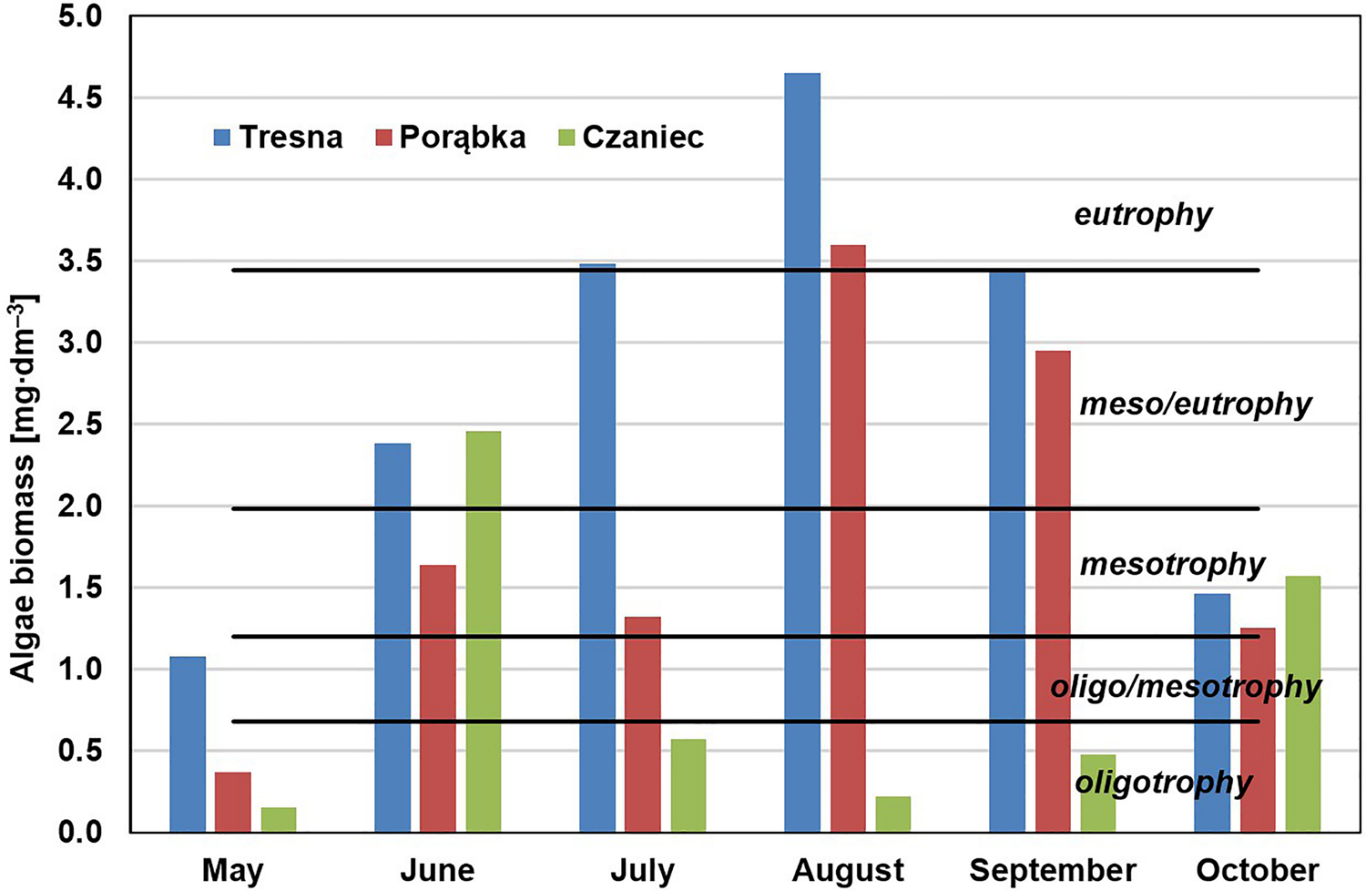
The planktonic algae biomass in water of the Soła cascade reservoirs in 2019 year and the trophic classification of Heinonen^41^.

The planktonic algae biomass in water is related to the condition of the aquatic environment^55^, i.e., temperature, light, nutrient availability, and water movement. Therefore, it can be very variable, which was confirmed by the results of this study (Fig. 4). The obtained average values of phytoplankton biomass were 2.75 mg∙dm^–3^ in the waters of the Tresna Reservoir, 1.86 mg∙dm^–3^ in the waters of the Porąbka Reservoir and 0.91 mg∙dml^–3^ in the waters of the Czaniec Reservoir. The biomass values varied greatly over the entire study period (Tresna: SD = 1.24; Porąbka: SD = 1.09; Czaniec: SD = 0.84). The Czaniec Reservoir was characterized by the greatest instability in phytoplankton development; on the one hand, it is shallow, which favors water heating, but on the other hand, it has high flow-through. The large phytoplankton biomass in the Czaniec Reservoir in June resulted mainly from the occurrence of a large number of *Dinophyceae* of the genus *Peridinium*, which are characterized by large cell dimensions.

The trophic state of the Tresna Reservoir, as determined by the planktonic algae biomass, changed in the individual series of measurements during the study period. It varied from a transition state between oligotrophy and mesotrophy to eutrophy, and the eutrophy was especially large in August. In the Porąbka Reservoir, the trophic states varied from oligotrophic to eutrophic; in the three series of measurements, it was mesotrophic. In turn, for the Czaniec Reservoir, 4 oligotrophic states, 1 mesotrophic and 1 transition state between mesotrophic and eutrophic were identified.

### Groups of planktonic algae

A preliminary assessment of aquatic environment fertility is possible on the basis of the dominance of a specific group of algae. For example, *Chrysophyceae* are a group of algae in which most species grow in waters that are poor in phosphorus and usually well oxygenated. They are most often found in the phytoplankton of oligotrophic lakes, often alpine and Finnish lakes. The presence of flagella and the capacity for mixotrophic feeding (alternating autotrophic and heterotrophic feeding) make it easier for them to be present in waters that are poor in phosphorus and are often under ice, where there is no light. However, some species can be found under different trophic conditions^56–60^. In contrast, large numbers of *Cyanobacteria* with high biomass indicate eutrophic conditions. Large colonies of *Cyanobacteria* of the genera *Microcystis* and *Woronichinia* are present in the waters of eutrophic reservoirs and form water blooms. They also secrete toxins, which are dangerous to humans and animals. Filamentous *Cyanobacteria* of the genera *Anabaena* and *Aphanizomenon* are also indicators of eutrophic conditions. They also create water blooms and secrete toxins^13,17,61–63^. An intensification in cyanobacterial blooms is a sign of advanced eutrophication^20^.

**Figure 5:**
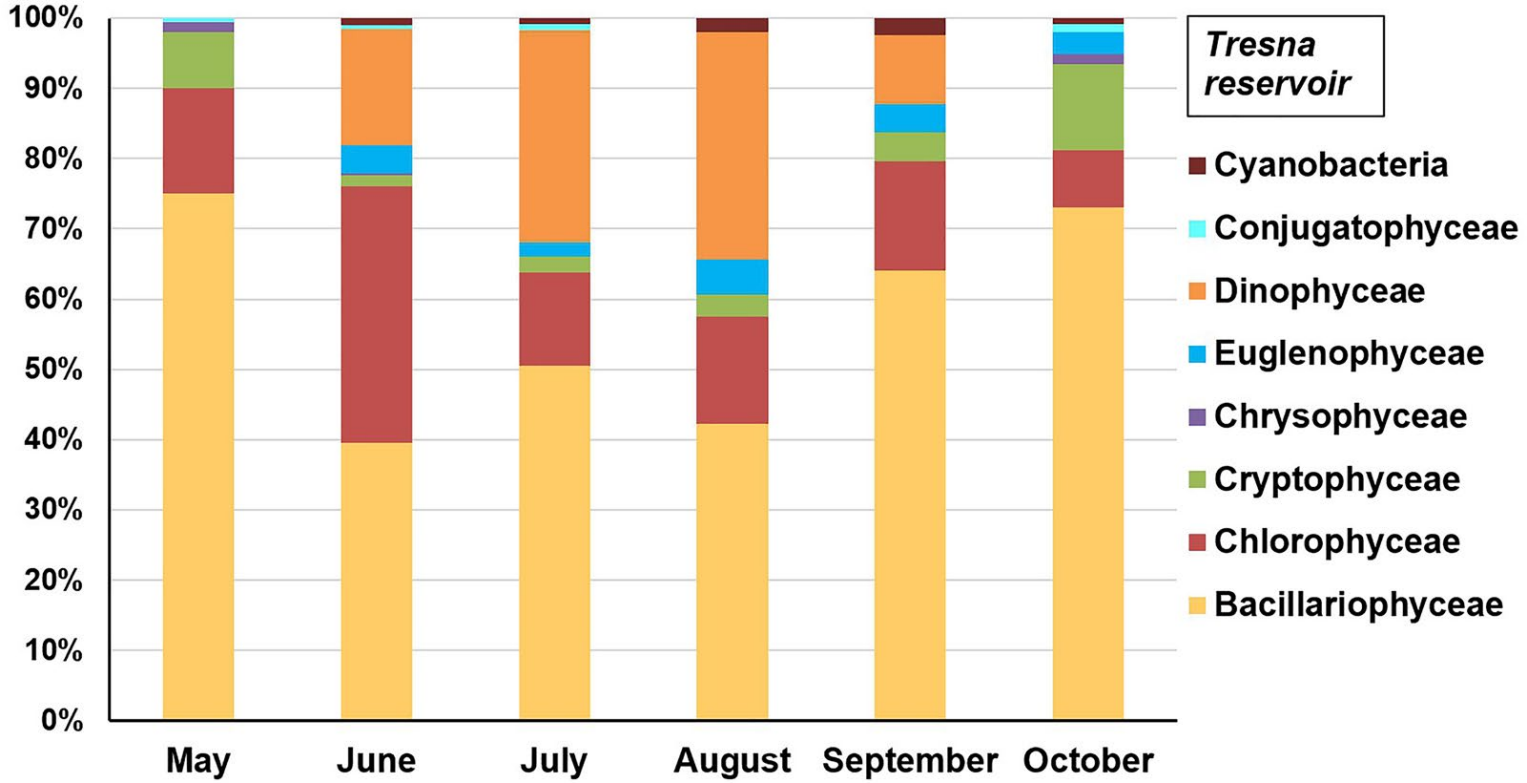
Structure of planktonic algae groups in the Tresna Reservoir in 2019 year.

**Figure 6:**
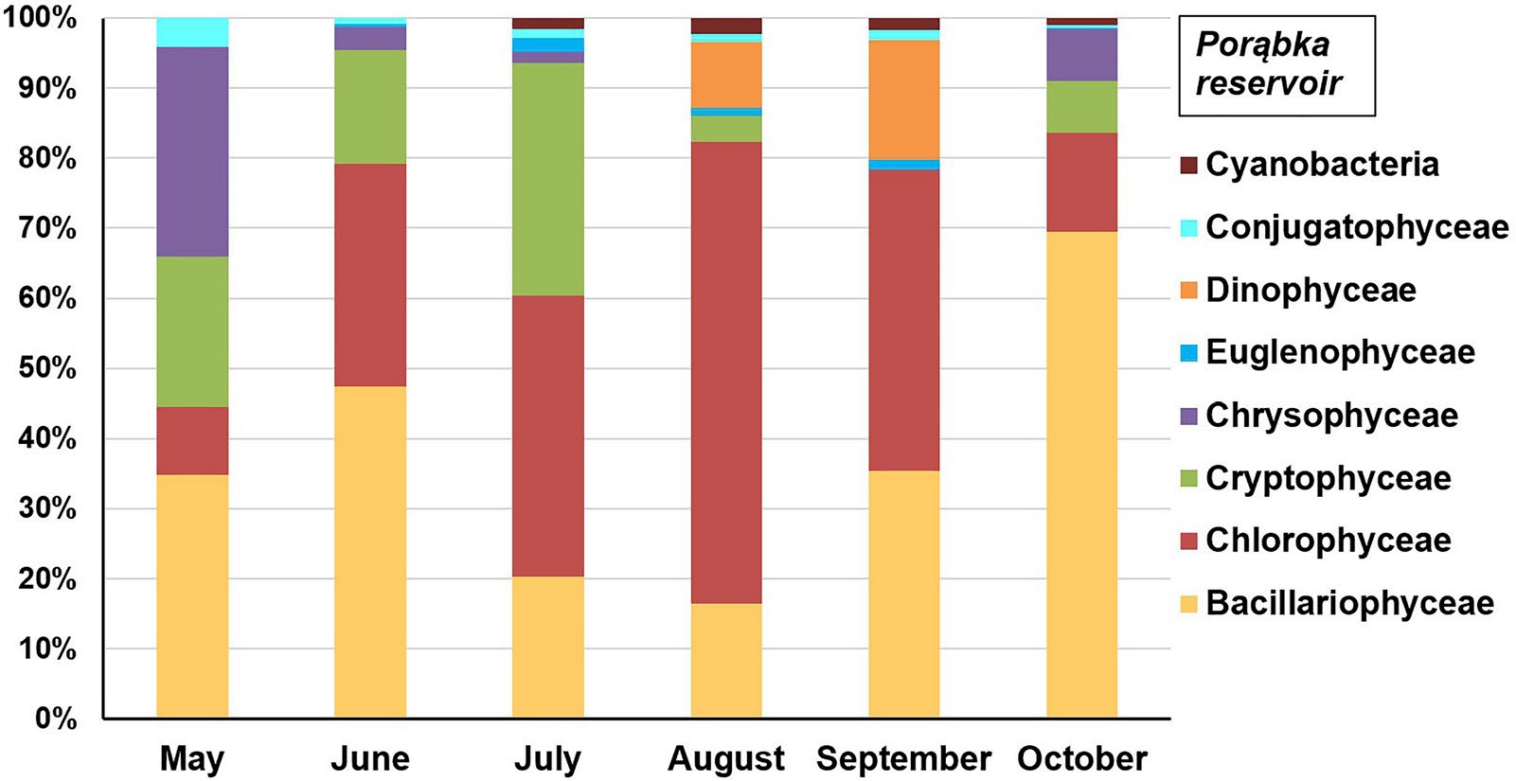
Structure of planktonic algae groups in the Porąbka Reservoir in 2019 year.

**Figure 7:**
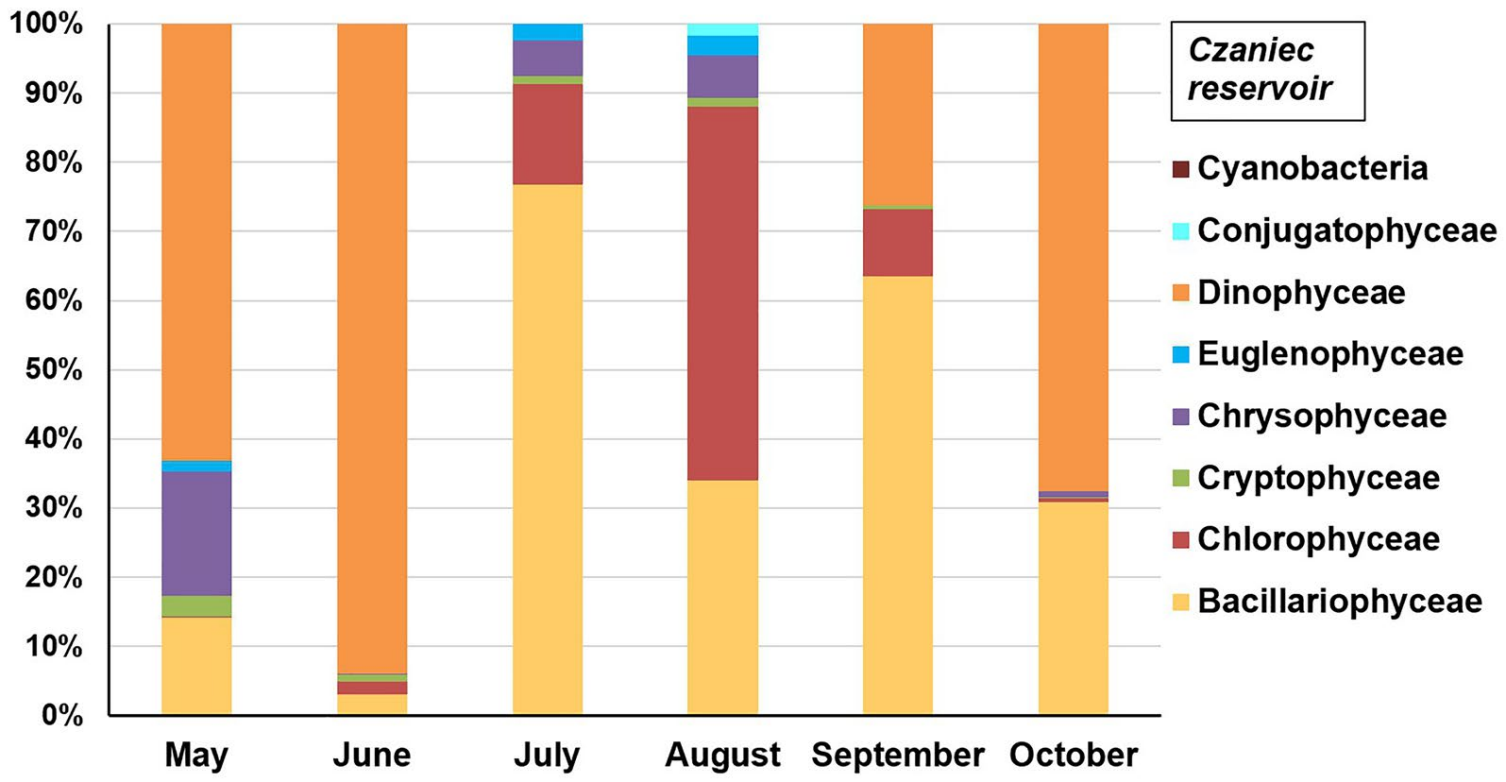
Structure of planktonic algae groups in the Czaniec Reservoir in 2019 year.

However, the structure of phytoplankton communities is rarely model structure to a degree sufficient to allow for the determination of accurate inferences about the trophic state of reservoirs (due to the high variability in species diversity, density and biomass of algae); therefore, researchers treat this parameter as a supporting parameter^10,15,64–66^. The results of the current study showed very high variability in the phytoplankton structure of these reservoirs during the period from May to October (Figs. 5, 6, 7).

Diatoms dominated the waters of the Tresna Reservoir throughout the study period (Fig. 5). Their share in the total phytoplankton biomass varied from 39.4% (June) to 75% (May). The greatest number of diatoms was observed in spring and autumn because they developed best during the period of water circulation in the reservoir. The heavy silica shells surrounding the diatom cells caused them to sink toward the bottom sediments during the water stagnation periods. Green algae constituted the second most significant group of algae. Their largest share was recorded in June—it amounted to 36.5%. The share of green algae in the remaining months varied between 8.2% (October) and 15.5% (September). Green algae genera predominated, which are characteristic of eutrophic environments, i.e., *Pediastrum, Coelastrum*^67^. Dinoflagellates, mainly *Ceratium hirundinella* species, also appeared in significant amounts during the period from June to September. The increasing content of mineral nitrogen in the water causes that *Ceratium hirundinella* gives place to the blue‒green algae *Microcystis aeruginosa*^68^. The shares of dinoflagellates in July and August amounted to 30.1% and 32.3%, respectively. An insignificant share of euglenins and *Cyanobacteria* were also recorded in the reservoir water. No large forms of colonial *Cyanobacteria* were found, which are indicators of advanced eutrophication. This may indicate that the reservoir was not strongly eutrophic, but there were periodic increases in water fertility to the eutrophic level.

The biomass of diatoms and green algae significantly exceeded those of other groups of algae in the Porąbka Reservoir (Fig. 6). The share of diatoms in the overall biomass changed from 16.4% in August to 69.5% in October, while green algae changed from 9.7% in May to 65.8% in August. The highest share of diatoms was observed in spring and autumn, as in the Tresna Reservoir. The green algae developed particularly strongly during the summer because their growth is favored by sunlight and high temperatures. Cryptophytes achieved a significant share in the period from May to July, with 33.3% in July. Dinoflagellates in the Porąbka Reservoir had a much smaller share in the biomass than in the Tresna Reservoir. They occurred only in August (9.4%) and September (17.1%). A large share of *Chrysophyceae* was found (29.9%) in the first month of the study. They develop most often in waters that are poor in phosphorus, well-oxygenated and oligotrophic^57–59^. The share of euglenins and *Cyanobacteria* within the overall algal biomass was negligible and did not exceed 2.5%. Similar to the Tresna Reservoir, no large forms of *Cyanobacteria* were observed (e.g., from the genus *Microcystis*), which would indicate the advanced eutrophication of reservoir water. In contrast, euglenins usually develop in waters that are rich in organic matter^68,69^. This may indicate that the reservoir periodically takes on the features of a eutrophic reservoir and of an oligotrophic reservoir because the algal characteristic of all trophic states were observed during the research season.

In the waters of the Czaniec Reservoir, small forms of phytoplankton usually dominated, which are characteristic of turbulent and unstable conditions; it is a high-flow reservoir. The only large-sized algae were dinoflagellates of the genus *Peridinium,* which mainly influenced the size of the phytoplankton biomass in the water of this reservoir. They had an advantage over other groups at the beginning and end of the study period (Fig. 7). Diatoms were the second most significant group of algae, with an average share of 37.1% (maximum 76.8% in July). The largest share of green algae was observed in August (54%). A significant percentage of *Chrysophyceae* was found*,* especially in May (18%). *Euglenophyceae, Cryptophyceae and Coniugatophyceae* accounted for a small share of the overall biomass of phytoplankton. The share of any of these groups did not exceed 3%. *Cyanobacteria* were not found in the waters of the reservoir, which may indicate that the water of the reservoir had poor fertility (small amounts of biogenic compounds and organic matter).

### Indicator species

Species, and alternatively the genera of planktonic algae, are used by many researchers for the evaluation of trophic state^13,15,17,21,58,64,70,71^. The so-called indicator algae are the most important. They have a narrow range of ecological tolerance and usually occur under conditions consistent with a single trophic state^19,72^. Many indicator algae were identified in the studied reservoirs of the Soła cascade (Table 2), which indicated a differentiation of trophy in the individual reservoirs. Single species, even indicator species, appeared under uncharacteristic trophic conditions. In such cases, attention was given to the number of individual algae and their biomass. The so-called ubiquitous algae also occurred in the water samples. They have wide ecological tolerance and live in various trophic and environmental conditions.

**Table 2:** The indicator algae in the reservoirs of the Soła cascade in 2019 year, characteristic for the conditions of eutrophy (e), mesotrophy (m), oligotrophy (o)—sorted by number of algae (from largest to smallest).

Month	Tresna reservoir	Porąbka reservoir	Czaniec reservoir
May	*Fragilaria crotonensis* (e) *Fragilaria capucina* (e) *Hipodonta capitata* (e) *Diatoma vulgaris* (e) *Asterionella formosa* (e) *Encyonema minutum* (o) *Amphora ovalis* (e) *Gyrosigma* sp. (e) *Melosira varians* (e)	*Encyonema minutum* (o) *Diatoma mesodon* (o) *Tabellaria fenestrata* (o, m) *Pinnularia* sp. (o) *Achnanthes lanceolata* (o) *Chrysococcus minutus* (o) *Gyrosigma* sp. (e) *Melosira varians* (e)	*Dinobryon divergens* (o) *Dinobryon cylindricum* (o) *Chrysococcus minutus* (o) *Tabellaria fenestrata* (o, m) *Diatoma mesodon* (o) *Achnanthes lanceolata* (o) *Pinnularia* sp. (o)
June	*Fragilaria capucina* (e) *Nitzschia acicularis* (e) *Scenedesmus quadricauda* (e) *Hipodonta capitata* (e) *Asterionella formosa* (e) *Diatoma vulgaris* (e) *Coelastrum microporum* (e) *Pediastrum boryanum* (e) *Pediastrum duplex* (e) *Gyrosigma* sp. (e) *Nitzschia sigmoidea* (m, e) *Amphora ovalis* (e)	*Encyonema minutum* (o) *Diatoma mesodon* (o) *Tabellaria fenestrata* (o, m) *Achnanthes lanceolata* (o) *Asterionella formosa* (e) *Dinobryon divergens* (o) *Botrycoccus braunii* (m, o) *Dinobryon cylindricum* (o) *Chrysococcus minutus* (o) *Crucigenia tetrapedia* (e) *Pediastrum duplex* (e) *Pediastrum boryanum* (e) *Gyrosigma* sp. (e)	*Diatoma mesodon* (o) *Encyonema minutum* (o) *Tabellaria fenestrata* (o, m) *Chrysococcus minutus* (o) *Achnanthes lanceolata* (o) *Dinobryon divergens* (o) *Pinnularia* sp. (o)
July	*Fragilaria crotonensis* (e) *Nitzschia acicularis* (e) *Scenedesmus quadricauda* (e) *Scenedesmus acutus* (e) *Fragilaria capucina* (e) *Aulacoseira granulata* (e) *Tetraedron minimum* (e) *Crucigenia tetrapedia* (e) *Anabaena flos-aquae* (e) *Oocystis borgei* (m) *Pediastrum boryanum* (e) *Pediastrum duplex* (e) *Nitzschia sigmoidea* (m, e) *Closterium aciculare* (e)	*Oocystis borgei* (m) *Scenedesmus quadricauda* (e) *Pediastrum duplex* (e) *Coelastrum microporum* (e) *Crucigenia tetrapedia* (e) *Pediastrum boryanum* (e) *Diatoma vulgaris* (e) *Asterionella formosa* (e) *Nitzschia acicularis* (e) *Hipodonta capitata* (e) *Fragilaria crotonensis* (e) *Fragilaria capucina* (e) *Aulacoseira granulata* (e) *Amphora ovalis* (e) *Nitzschia sigmoidea* (m, e)	*Diatoma mesodon* (o) *Encyonema minutum* (o) *Monoraphidium komarkovae* (o, m) *Pinnularia* sp. (o) *Achnanthes lanceolata* (o) *Asterionella formosa* (e) *Scenedesmus* sp. (e)
August	*Fragilaria crotonensis* (e) *Nitzschia acicularis* (e) *Scenedesmus quadricauda* (e) *Scenedesmus acutus* (e) *Tetraedron caudatum* (e) *Crucigenia tetrapedia* (e) *Crucigeniella apiculata* (e) *Aulacoseira granulata* (e) *Pediastrum duplex* (e) *Pediastrum boryanum* (e) *Oocystis borgei* (m) *Anabaena flos-aquae* (e) *Nitzschia sigmoidea* (m, e) *Amphora ovalis* (e) *Closterium aciculare* (e)	*Scenedesmus quadricauda* (e) *Oocystis borgei* (m) *Botrycoccus braunii* (m, o) *Pediastrum tetras* (e) *Pediastrum boryanum* (e) *Aulacoseira granulata* (e) *Asterionella formosa* (e) *Fragilaria crotonensis* (e) *Fragilaria capucina* (e) *Diatoma vulgaris* (e) *Hipodonta capitata* (e) *Nitzschia acicularis* (e) *Crucigenia tetrapedia* (e) *Nitzschia sigmoidea* (m, e) *Amphora ovalis* (e)	*Encyonema minutum* (o) *Monoraphidium komarkovae* (o, m) *Tabellaria fenestrata* (o, m) *Achnanthes lanceolata* (o) *Tetraedron minimum* (e) *Scenedesmus* sp. (e) *Nitzschia sigmoidea* (m, e)
September	*Fragilaria crotonensis* (e) *Hipodonta capitata* (e) *Asterionella formosa* (e) *Aulacoseira granulata* (e) *Scenedesmus acutus* (e) *Scenedesmus quadricauda* (e) *Crucigenia tetrapedia* (e) *Crucigeniella apiculata* (e) *Anabaena flos-aquae* (e) *Amphora ovalis* (e) *Gyrosigma* sp. (e) *Oocystis borgei* (m)	*Pediastrum tetras* (e) *Pediastrum boryanum* (e) *Oocystis borgei* (m) *Diatoma mesodon* (o) *Botrycoccus braunii* (m, o) *Asterionella formosa* (e) *Chrysococcus minutus* (o) *Tabellaria fenestrata* (o, m) *Pinnularia* sp. (o) *Dinobryon divergens* (o) *Melosira varians* (e) *Achnanthes lanceolata* (o) *Encyonema minutum* (o) *Gyrosigma* sp. (e)	*Encyonema minutum* (o) *Dinobryon divergens* (o) *Diatoma mesodon* (o) *Achnanthes lanceolata* (o) *Dinobryon cylindricum* (o) *Chrysococcus minutus* (o) *Monoraphidium komarkovae* (o, m) *Tabellaria fenestrata* (o, m) *Tetraedron minimum* (e) *Scenedesmus* sp. (e) *Asterionella formosa* (e) *Pinnularia* sp. (o)
October	*Diatoma vulgaris* (e) *Hipodonta capitata* (e) *Asterionella formosa* (e) *Melosira varians* (e) *Encyonema minutum* (o) *Scenedesmus acutus* (e) *Scenedesmus* sp. (e) *Crucigeniella apiculata* (e) *Crucigenia tetrapedia* (e) *Gyrosigma* sp. (e)	*Encyonema minutum* (o) *Diatoma mesodon* (o) *Tabellaria fenestrata* (o, m) *Chrysococcus minutus* (o) *Dinobryon divergens* (o) *Dinobryon cylindricum* (o) *Achnanthes lanceolata* (o) *Pinnularia* sp. (o) *Pediastrum boryanum* (e) *Pediastrum tetras* (e) *Melosira varians* (e)	*Diatoma mesodon* (o) *Encyonema minutum* (o) *Tabellaria fenestrata* (o, m) *Achnanthes lanceolata* (o) *Dinobryon divergens* (o) *Chrysococcus minutus* (o) *Pinnularia* sp. (o) *Dinobryon cylindricum* (o)

In the Tresna Reservoir, diatoms were the dominant group of algae. Notably, species characteristic to eutrophic waters were observed, i.a. *Fragilaria crotonensis, Aulacoseira granulata, Nitzschia acicularis, Asterionella formosa, Diatoma vulgaris*. These taxa have been observed in eutrophic reservoirs in various parts of the world—Finnish lakes^60^, Czech reservoirs^73^ and Chinese^23^, as well as in Polish oxbow lakes rich in biogens^74^. Species with oligotrophic (e.g., *Encyonema minutum*) and mesotrophic preferences (e.g., *Oocystis borgei*) also appeared in small numbers.

Notably, a large number of diatoms of the species *Fragilaria crotonensis* (often over 2800 cells in 1 cm^3^) were present, which indicate eutrophication processes. For a water bloom in reservoirs bloom is considered to be the presence of algae in the number of over 500, alternatively more than 1000 cells per 1 cm^3^ of water^75^. *Fragilaria crotonensis* generally occurs in mesotrophic and eutrophic waters with high electrolyte content, while intensive development takes place under eutrophic conditions^44^. The eutrophic preferences of *Fragilaria crotonensis* have been confirmed in many studies^63,76,77^. Znachor et al.^73^ observed this species together with *Asterionella formosa* during periods of high water phosphorus and nitrogen content. Çelekli and Öztürk^64^ confirmed that the taxa of the genus *Fragilaria* prefer higher contents of phosphate in waters.

The periodic and intensive development of the diatom taxon of *Nitzschia acicularis* (often over 500 cells in 1 cm^3^ of water) also indicated the eutrophication of the Tresna Reservoir. This taxon lives in poorly and strongly eutrophic waters with a high content of electrolytes. According to Yilmaz et al.^21^, this taxon is sensitive to nutrient deficiencies. Algae typical to eutrophic waters also dominated among green algae, i.a., *Pediastrum* sp., *Scenedesmus* sp., *Coelastrum* sp. In the opinion of Herrmann^78^, the development of these green algae indicates the eutrophy of a reservoir. Berlinger and Sigee^67^ also consider these green algae typical for eutrophic conditions. They have been observed in many eutrophic reservoirs, e.g., in the Portuguese lake of Braças^79,80^.

It is worth adding that *Cyanobacteria* were present in the Tresna Reservoir, mainly of the genera *Oscillatoria*, *Lyngbya*, *Merismopedia* and species of *Anabaena flos-aqae,* which have been previously described in eutrophic Polish reservoirs^62^ and Italian lakes^17^. However, large colonial *Cyanobacteria* typical of strongly eutrophic waters were not observed, such as *Microcystis* sp. and *Aphanizomenon* sp., but the phytoplankton species composition of the reservoir indicated eutrophy because many of them are typical for eutrophic waters.

Indicator algae varied over the study period in the Porąbka Reservoir. Species characteristic of oligotrophic environments dominated in May, June and October (e.g., *Encyonema minutum, Diatoma mesodon, Achnanthes lanceolata*, *Tabelaria fenestrata*). Keatley et al.^81^ reported that *Achnanthes lanceolata, Tabelaria* sp., and *Encyonema minutum* prefer oxygenated oligotrophic and oligomesotrophic water. The diatoms *Diatoma mesodon* and *Pinnularia* sp. have also been often observed in less fertile waters, e.g., in oligotrophic subalpine water reservoirs^82^, as well as in mountain lakes in the Pyrenees^83^. Wentzky et al.^84^ found the presence of diatoms of *Tabelaria fenestrata* after a reduction in the concentration of biogenic substances in the water supply to the Rappbode reservoir in the Harz Mountains of Germany.

Species characteristic to eutrophic and mesotrophic environments developed in the Porąbka Reservoir over the summer, i.e., in July and August. During that time, the green algal species *Oocystis borgei,* which is characteristic of mesotrophic waters, accounted for a large share^44^. September was the month in which species consistent with all trophic environments occurred; the most abundant taxa were related to eutrophy. The reservoir therefore has a variable character—it periodically shows features of high fertility and low fertility. It may be suggested that this reservoir may have intermediate trophic features that are in between those of the Tresna and Czaniec reservoirs because typical eutrophic algae were not as abundant as in the Tresna Reservoir and they also did not attain a large biomass, but algal taxa typical to oligotrophic reservoirs were observed less frequently than in the Czaniec Reservoir.

In the Czaniec Reservoir, small diatoms and green algae were mainly present, which quickly adapted to changes in the environmental conditions. These forms are most often dominant in lakes and reservoirs that are poor in nutrients, as previously reported in studies from various countries^15,66,82,83,85,86^. Algae indicating oligotrophic states prevailed during all study periods, but a sparse occurrence of species preferring fertile environments was found from July to September.

Among the algal characteristic of clean water (poor in nutrients) observed in the Czaniec Reservoir, it is worth mentioning that *Achnanthes lanceolata, Tabellaria* sp., *Encyonema minutum*, *Diatoma mesodon,* and *Pinnularia* sp. *Chrysophyceae* were also common, especially the genus *Dinobryon.* They belong to mixotrophs (periodically they can feed in a heterotrophic manner, that is, in an animal way) and have the ability to catch and digest small food particles, e.g., bacteria. This ability serves as an alternative food strategy during nutrient deficient conditions. The ability to move makes it easier for them to migrate toward optimal development conditions^57,58^. *Dinobryon divergens* that occurred in the Czaniec Reservoir are characteristic of small and shallow water reservoirs that are poor in nutrients^64^. The Czaniec Reservoir can be represented as oligotrophic with the potential for periodic increases in fertility, depending on external factors.

### Total phosphorus

Phosphorus is an important indicator of the fertility of aquatic ecosystems^87–89^. The availability of phosphorus determines the growth of algae and plant biomass because it is an element that limits their development (the ratio of C:N:P for algae is 40:7:1). It is the most eutrophogenic element. As Vollenweider^90^ reported, massive growth of algae (a water bloom) can occur at a total water phosphorus concentration of 0.015 mg∙dm^–3^. The total phosphorus content is an indicator of water fertility. However, planktonic algae absorb phosphates, condensing them during enzymatic reactions into polyphosphates.

**Figure 8:**
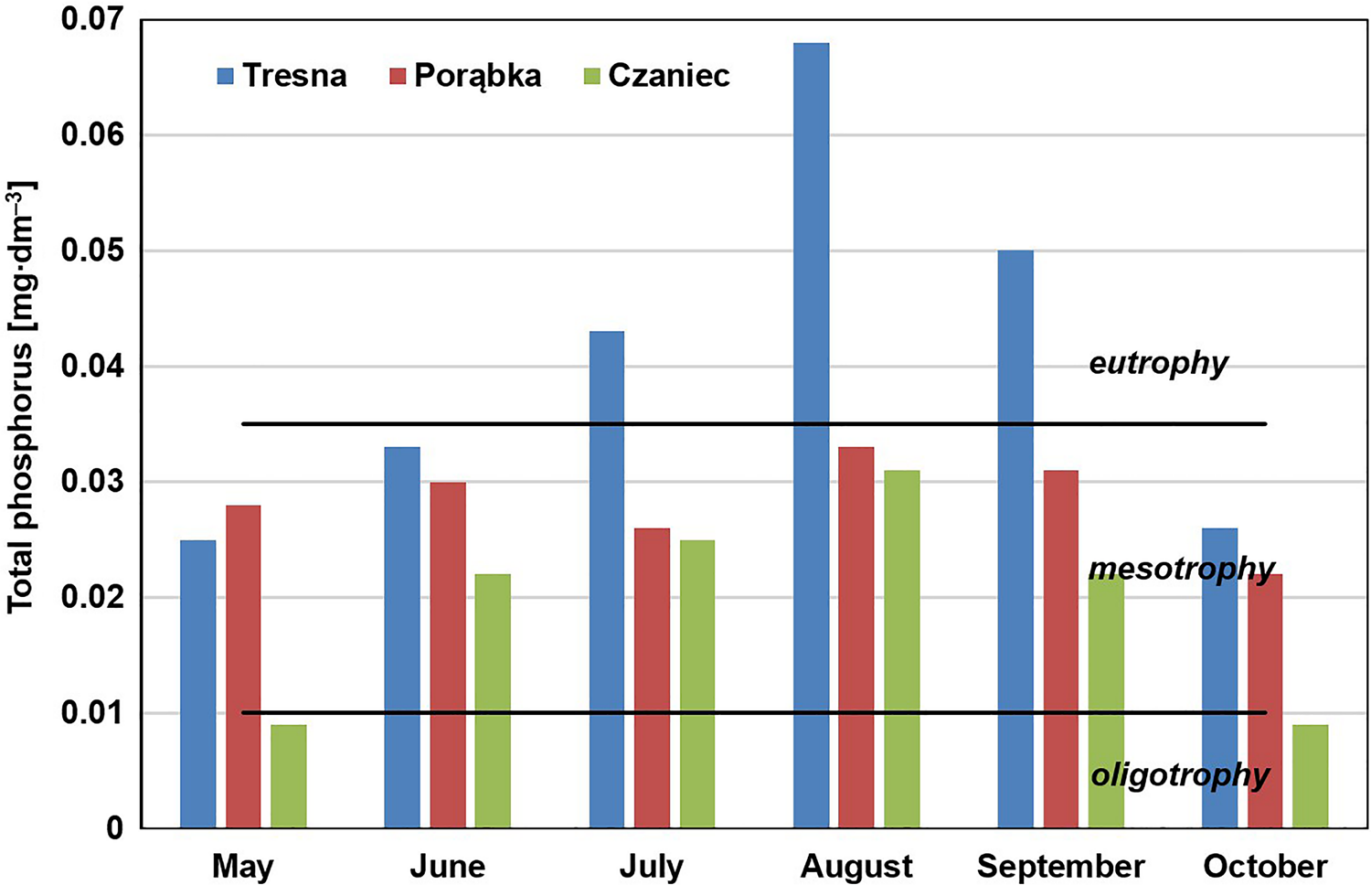
Concentrations of the total phosphorus in water of the Soła cascade reservoirs in 2019 year and the OECD trophic classification^11^.

The total phosphorus concentrations varied and were localized within the ranges of the different trophic states (Fig. 8). They varied from 0.025 to 0.068 mg∙dm^–3^ in the Tresna Reservoir; these concentrations indicated mesotrophy at the beginning and at the end of the study period, while they indicated eutrophy during the entirety of the summer (July, August, September). In the Porąbka Reservoir, the concentration of total phosphorus in all measurement series was within the range that represents mesotrophy. The total phosphorus concentration did not exceed 0.01 mg∙dm^–3^ in the first (in May) and in the last (in October) series of measurements in the Czaniec Reservoir, which indicated oligotrophy, while in the remaining series, it was characteristic of mesotrophy.

The phosphorus supplied the Soła cascade reservoirs is used in biological production, but is also precipitated to bottom sediments^91^. Higher concentrations of phosphorus in reservoir water testify to the accumulation of large amounts of phosphorus during several dozen years of exploitation of these reservoirs. Currently, phosphorus is likely subject to circulation in reservoir ecosystems. The slight self-purifying effect is visible—the concentrations of the phosphorus decrease slightly in the subsequent reservoirs in the cascade; however, in every case, they are enough that they can cause water blooms.

### Integral Trophic State index (ITS)

The Integral Trophic State index (ITS), which was elaborated by Neverowa-Dziopak^4,46^ and has been tested in various aquatic environments^92–95^, is calculated based on measurements of pH and water oxygen saturation. The pH values varied from 6.95 to 8.60 (average of 7.44) in the Tresna Reservoir. The pH values were lower in the Porąbka Reservoir, from 6.72 to 7.50 (average of 6.99), and they were the lowest in the Czaniec Reservoir, from 6.57 to 7.32 (average of 6.90). This may indicate a loss of carbonates during the flow of water through the cascade. The oxygen saturation of water varied from 80.4 to 140.6% (average of 107%) in the Tresna Reservoir, from 80.4 to 122.6% (average of 100.3%) in the Porąbka Reservoir and from 92.3 to 149.4% (average of 124.9%) in the Czaniec Reservoir. The correlation between pH and oxygen saturation is a necessary condition for the calculation of the ITS; therefore, correlation analysis was performed for n = 6 and α = 0.05. The results of this analysis are presented in Table 3. Very high correlation coefficients were obtained despite the small number of measurement series. The correlation between the tested parameters was statistically significant.

**Table 3:** Correlation analysis between pH and oxygen saturation for the water of the Soła cascade in 2019.

Correlation factors	Tresna reservoir	Porąbka reservoir	Czaniec reservoir
Correlation coefficient *r*	0.961	0.953	0.958
Coefficient of determination *R*^*2*^	0.925	0.908	0.918
Correlation relevance	Yes	Yes	Yes
Linear regression coefficient *a*	0.024	0.019	0.010
Linear regresion relevance	Yes	Yes	Yes

ITS calculations gave the following results:

- the Tresna Reservoir—7.515,
- the Porąbka reservoir—7.070,
- the Czaniec reservoir—6.722.

The decrease in the ITS value in the successive reservoirs of the cascade testifies to the reduced potential for biological production. According to the ITS trophic scale^4,46^, the Tresna Reservoir was classified as mesotrophic, while the Porąbka and Czaniec Reservoirs were classified as oligotrophic reservoirs. The ITS value for the Czaniec Reservoir was close to that of ultraoligotrophy; as mentioned earlier, the water flows into this reservoir, which undergoes the self-purifying in the Tresna and Porąbka Reservoirs. In this reservoir, sediment accumulation does not occur due to the short retention time of water.

### Summary of the results of trophic classification

The criteria used to determine the trophic state of individual reservoirs of the Soła cascade were varied and did not always allow for unequivocal conclusions. The difficulties with the assessment of the trophic states resulted from the high variability of values of the studied parameters. Attempts to compile the trophic classifications are shown in Table 4, in which the following principles were applied:

- in parameterized criteria (with ranges of values), the number of months was recorded at a defined trophic state during which this state was found;
- in the case of ITS, one result was valid for the overall study period;
- in the qualitative criteria for phytoplankton, the high dynamic of the organisms changes was taken into account, and trophic states were assigned.

**Table 4:** Trophic states of the Soła cascade reservoirs in 2019 year.

Classification criterion	Tresna reservoir	Porąbka reservoir	Czaniec reservoir
Chlorophyll *a* concentration	Mesotrophy (4) Eutrophy (2)	Oligotrophy (2) Mesotrophy (4)	Oligotrophy (4) Mesotrophy (2)
Planktonic algae biomass	Oligo/mesotrophy (1) Mesotrophy (1) Meso/eutrophy (2) Eutrophy (2)	Oligotrophy (1) Mesotrophy (3) Meso/eutrophy (1) Eutrophy (1)	Oligotrophy (4) Mesotrophy (1) Meso/eutrophy (1)
Total phosphorus concentration	Mesotrophy (3) Eutrophy (3)	Mesotrophy (6)	Oligotrophy (2) Mesotrophy (4)
ITS	Mesotrophy	Oligotrophy	Oligotrophy
Planktonic algae groups	Mesotrophy Eutrophy	Oligotrophy Eutrophy	Oligotrophy
Indicator algae	Eutrophy	Oligotrophy Eutrophy	Oligotrophy

A comparison of the trophic state assessment according to various criteria shows that depending on the adopted criterion, the different results may be obtained. For example, the Tresna Reservoir had the following trophic state in June: based on the content of chlorophyll *a* – eutrophy, based on the algae biomass – meso/eutrophy, based on the content of total phosphorus – mesotrophy. In turn, the trophic state of Porąbka Reservoir in September was oligotrophy (based on the content of chlorophyll *a*), meso/eutrophy (based on the algae biomass) or mesotrophy (based on the content of total phosphorus). The fewer differences in assessment concerned the Czaniec Reservoir, which is characterized by lower amplitudes of flows and less fertility of water.

The comparison shows that the Tresna Reservoir is characterized by the highest fertility of the entire cascade. It shows features of a eutrophic or mesotrophic reservoir. It appears that this reservoir should be assigned a transitional trophic state between mesotrophic and eutrophic. The Porąbka Reservoir is the most trophically unstable because all trophic states were found in the study. The mesotrophic state was most often identified in the parameterized criteria, but periodically, the reservoir had an oligotrophic character. The trophic state of this reservoir can be considered a transitional state between mesotrophic and oligotrophic. The Czaniec Reservoir most often showed oligotrophic features, and it should be classified at this level. However, it is worth adding that this reservoir has the potential for biological production due to the abundance of phosphorus.

## Conclusions

The dam reservoirs of mountainous areas can be characterized by a high variability of trophic parameters, which the studies of the Soła cascade have shown. The features of the phytoplankton species structure were particularly unstable. The trophic states of individual reservoirs of the Soła cascade were difficult to unequivocally define.

The trophic state of the same reservoir was often assessed differently depending on the criterion used. This indicates the need for methodological research.

Biological production of phytoplankton has proved to be a good indicator of trophic conditions, despite the high quantitative and qualitative dynamics during the vegetative season. The features of phytoplankton in the reservoirs of the Soła cascade clearly corresponded with dynamics of the fertility of aquatic ecosystems.

The Soła cascade has a purifying role for water resources, which is expressed as a reduction in the fertility of the environment of subsequent reservoirs. The catchment area of the Tresna Reservoir should be covered by a special program for water resource protection to limit of the nutrients migration and eutrophication of this reservoir.

## Data Availability

The datasets used during the current study are available from the corresponding or first author on reasonable request.
